# Genome-Wide Association Study and Genomic Selection for Average Daily Gain in Ashidan Yak

**DOI:** 10.3390/ani16162452

**Published:** 2026-08-07

**Authors:** Zhicheng Wang, Xiaoming Ma, Guangwei Hu, Jianwu Jing, Yongfu La, Wenwen Ren, Baicheng Zhou, Hongkang Li, Min Chu, Xiaoyun Wu, Ping Yan, Xian Guo, Chunnian Liang

**Affiliations:** 1 Key Laboratory of Yak Breeding Engineering of Gansu Province, Lanzhou Institute of Husbandry and Pharmaceutical Sciences of Chinese Academy of Agricultural Sciences, Lanzhou 730046, China; 18612203830@163.com (Z.W.); maxiaoming@caas.cn (X.M.); layongfu@caas.cn (Y.L.); rww15806106435@126.com (W.R.); chumin@caas.cn (M.C.); wuxiaoyun@caas.cn (X.W.); pingyanlz@163.com (P.Y.); guoxian@caas.cn (X.G.); 2 Key Laboratory of Animal Genetics and Breeding on Tibetan Plateau, Ministry of Agriculture and Rural Affairs, Lanzhou 730050, China; 3 Qinghai Province Yak Breeding and Promotion Service Center, Xining 810100, China; qhhgw@sina.com (G.H.); qmzyjjw@163.com (J.J.); baichengzhou2026@126.com (B.Z.); hongkangli2026@126.com (H.L.)

**Keywords:** Ashidan yak, average daily gain, whole-genome resequencing, genome-wide association study, genomic selection, candidate genes

## Abstract

Average daily gain (ADG) is a core quantitative trait determining the economic benefits of Ashidan yak, a polled new breed adapted to cold barn feeding on the Qinghai–Tibet Plateau. Unraveling its complex genetic architecture is crucial for early molecular breeding selection. In this study, high-depth whole-genome resequencing (WGS) data from 474 Ashidan yaks were used to conduct combined evaluation of genome-wide association study (GWAS) and genomic selection (GS). During GWAS analysis, sex and measurement batch were included as fixed effects, while birth weight and principal components (PC1–PC3) were incorporated as covariates. Multi-model association analysis using GLM, MLM, and FarmCPU was performed on 3.36 million LD-pruned SNPs. The genomic inflation factors (λ ≈ 1.0) for MLM and FarmCPU confirmed effective elimination of population stratification. A total of 11 genome-wide significant SNP loci and 7 key candidate genes including *PDE10A*, *RAD51B*, *BCAS3* and *KCNH8* were identified via the FarmCPU model. Functional enrichment analysis indicated that these gene clusters are significantly involved in cAMP signaling pathway, regulation of ion channel activity, as well as extracellular matrix remodeling of blood vessels and skeletal muscle cells. For genomic selection, a single-trait GBLUP model was constructed using 22.87 million high-density raw SNPs to fully capture polygenic minor effects. Moderately high narrow-sense genomic heritability of ADG was estimated at h^2^ = 0.3233 (*p* < 0.05). The average independent prediction accuracy across the 10-fold cross-validation reached an average of R = 0.14 ± 0.07. To evaluate marker prioritized genomic evaluation without data leakage, a strict 10-fold cross-validation scheme was implemented, where the top 1% high-priority variant set (~228,000 SNPs) was screened independently within each training fold. The resulting unbiased prediction accuracy reached R = 0.1328 ± 0.1566 (with an average RMSE of 0.3793 ± 0.0066 and a regression slope of 0.4183 ± 0.5055). Comparing this with the unselected whole-genome baseline (R = 0.14 ± 0.07) indicates that naive marker selection based solely on GBLUP effect size in small reference cohorts is influenced by sampling variance, highlighting the need to integrate multi-omics functional annotations for future custom breeding array development. This study provides quantitative insights into the polygenic architecture of ADG in yaks, offering baseline data for genomic selection and custom array development for indigenous livestock on the Qinghai–Tibet Plateau.

## Simple Summary

The Ashidan yak is a unique polled breed developed for indoor farming on the Qinghai–Tibet Plateau. For herders, the daily growth rate of these yaks is the most critical factor for economic profitability. However, the genetic mechanisms controlling this growth remain complex and unclear. In this study, we analyzed the complete DNA sequences of 474 Ashidan yaks monitored from birth to six months of age to uncover the genetic basis of average daily gain (ADG) and test if their DNA can predict future weight gain using multi-model GWAS and genomic prediction. We discovered specific genes that help yaks manage energy, control appetite, and build muscle under harsh plateau conditions. Furthermore, we demonstrated that a yak’s growth potential can be reliably predicted early in life using its overall genetic profile. By pinpointing the most valuable top 1% genetic markers, we laid the groundwork for developing affordable DNA testing arrays. This means breeders can use a simple DNA test to select the fastest-growing yaks at birth, accelerating genetic improvement, increasing farmers’ incomes, and promoting sustainable livestock production in extreme environments.

## 1. Introduction

Yak (*Bos grunniens*) is a foundational species in the Qinghai–Tibet Plateau and its adjacent alpine ecosystems, supplying essential resources such as meat, milk and wool for over 17 million local herdsmen [1,2]. Nevertheless, traditional horned yak breeds raised under harsh natural grazing generally suffer from long growth cycles, low feed conversion efficiency, and seasonal body condition fluctuations featuring summer fattening, winter emaciation, and spring mortality [3,4]. To address this industrial bottleneck, Chinese researchers have independently developed the first polled yak breed, Ashidan yak, after years of breeding efforts [5,6]. This breed features gentle temperament, strong stress resistance and excellent adaptability to barn and intensive farming, representing an important development in the modernization of the yak industry [7]. In modern intensive beef production systems, average daily gain (ADG) serves as the pivotal quantitative trait directly determining feed conversion efficiency, slaughter age and overall economic benefits [8,9]. Therefore, in-depth exploration of the molecular genetic mechanism underlying ADG and establishment of early molecular prediction models are essential for sustainable genetic improvement of Ashidan yak [10]. Animal growth and development is a typical complex polygenic trait precisely regulated by intricate gene networks [11]. Over the past decade, genome-wide association study (GWAS) has been widely adopted to identify major genes governing growth traits in cattle. Zhang et al. carried out multi-strategy GWAS on 1173 genotyped samples via the Illumina BovineHD BeadChip, and pinpointed the DCAF16-NCAPG region as the core genetic segment governing cattle average daily gain [12]. Zhuang et al. conducted a weighted single-step genome-wide association study (wssGWAS) in Chinese Simmental cattle and identified key candidate genes for birth weight, 12-month body weight, 18-month body weight, and average daily gain from birth to 12 months, including SQOR, TBCB, MYH10, and RLF [13]. Nevertheless, most previous studies relied on low-density SNP chips. Limited genomic coverage tends to conceal authentic causal mutations and frequently results in missing heritability [14]. The rapid popularization of whole-genome resequencing (WGS) in recent years has enabled the acquisition of millions of high-density variations at the population level, greatly improving the resolution of quantitative trait locus (QTL) fine mapping [15,16]. High-density markers often bring severe statistical biases. Conventional general linear model (GLM) tends to produce false positive results, while mixed linear model (MLM) may lead to over-correction of polygenic signals and eliminate minor polygenic effects [17]. Accordingly, the FarmCPU model based on iterative fixed and random effects has been developed [18]. This model dynamically eliminates background noise by iteratively incorporating pseudo-quantitative trait nucleotides as fixed effects, thereby substantially improving the detection power of minor-effect mutations underlying complex traits. Leveraging such high-precision models, several candidate genes associated with skeletal and muscle development have been identified in livestock species, including yaks [19,20].

Nevertheless, relying solely on GWAS to screen a small number of significantly associated loci is insufficient to fully dissect and utilize the overall genetic variation in growth traits. As a core technology for modern molecular breeding, genomic selection (GS) can estimate the genomic estimated breeding value (GEBV) of individuals using genome-wide markers, thereby effectively capturing abundant minor polygenic effects that are frequently overlooked by conventional GWAS [21]. Among them, the genomic best linear unbiased prediction (GBLUP) model based on the genomic relationship matrix has become a universal standard for livestock genetic evaluation due to its robust capability for variance component decomposition [22]. For yaks inhabiting alpine pastoral regions that suffer from difficult phenotypic measurement and long growth cycles, the application of GS technology can eliminate reliance on traditional phenotyping and enable accurate early selection at the juvenile stage, which possesses great economic value and industrial promotion significance [23,24].

Inspired by these advances, this study focused on the core growth trait ADG of Ashidan yaks and performed in-depth mining using high-depth whole-genome resequencing data from 474 individuals. Strict quality control and linkage disequilibrium pruning were conducted during GWAS to eliminate statistical false positives [25,26]. Subsequently, multi-model cross-validation was performed using GLM, MLM and FarmCPU to precisely identify key candidate genes and biological pathways regulating average daily gain in Ashidan yaks with minimal background noise. On this basis, to recover missing heritability and comprehensively evaluate breeding potential, we adopted the full set of high-density variants without LD dimensionality reduction in the genomic selection (GS) analysis, and applied the univariate GBLUP model to accurately estimate the narrow-sense genomic heritability of ADG. Finally, core marker panels with high predictive value were screened out by extracting the additive effect weights of all SNPs. This study aims to provide theoretical and technical insights for the development of a custom breeding chip for Ashidan yaks and the popularization of low-cost molecular breeding technologies in the future.

## 2. Materials and Methods

### 2.1. Experimental Animals and Phenotypic Data Collection

The experimental population consisted of 474 randomly selected Ashidan yaks, including 241 females and 233 males. All samples were collected at Datong Cattle Breeding Farm in Qinghai Province, China. To minimize management and environmental variance, all experimental yaks belonged to the same birth cohort, born during the spring calving season of 2025 (from April to June 2025) at Datong Cattle Breeding Farm. Calves grazed on natural alpine meadow together with their dams without supplemental feeding, ensuring consistent natural environmental conditions across the population. Individual body weight was recorded at birth and at 6 months of age, with an average measurement interval of 157.83 ± 14.83 days (ranging from 125 to 204 days). Individual ADG (kg/day) was calculated over this exact time interval for each animal. Calves grazed on natural alpine meadow together with their dams without artificial creep feeding, ensuring consistent natural environmental and nutritional conditions across the experimental population. Body weight was recorded from birth to six months of age, and the average daily gain was calculated accordingly. The recording and calculation formula for the core phenotypic trait average daily gain (ADG, kg/day) are presented as follows:

$$ADG=\frac{Final\;weight-Newborn\;weight}{Number\;of\;days\;tested}$$

Prior to genetic analysis, normality tests were performed on the ADG phenotypic data. Blood samples (5 mL) were collected from the jugular vein of each yak into vacuum blood collection tubes containing EDTA anticoagulant and stored at −20 °C. Genomic DNA was extracted using the TIANamp Blood DNA Kit (Tiangen Biotech, Beijing, China) following the manufacturer’s protocols. The OD260/280 ratio was measured with a NanoDrop spectrophotometer to assess DNA purity, while DNA concentration and integrity were accurately quantified using a Qubit fluorometer (Thermo Fisher Scientific, Waltham, MA, USA).

### 2.2. Whole-Genome Resequencing and Genotype Quality Control

Qualified DNA samples were used for library construction following the procedures including restriction enzyme digestion, random fragmentation, end repair, A-tailing, adapter ligation and purification. The constructed libraries were sequenced on the DNBSEQ-T7 high-throughput sequencing platform. Raw image data generated by sequencing were converted into raw reads via base calling. To guarantee the quality of subsequent bioinformatic analysis, raw reads were filtered as follows: reads containing adapter sequences were discarded; reads with more than 10% ambiguous bases (N) and paired reads with over 95% low-quality bases (Phred score ≤ 20) were also removed. The filtered high-quality clean reads were aligned to the reference genome (Bos_grunniens.LU_Bosgru_v3.0.dna.toplevel.fa) using BWA software (v.0.7.17) [27], and duplicates were removed from the alignment results with SAMTOOLS (v.1.9) [28]. Variant calling was performed on BAM files using the GATK HaplotypeCaller module (v.4.2.6.1) [29]. Individual genomic variant call format (GVCF) files were generated for each sample, which were then merged into a single file via CombineGVCFs. The combined variant file was subsequently genotyped using GenotypeGVCFs. The average sequencing depth across all samples was approximately 21.64×. The genome coverage reached 95.09% at 1×, 81.28% at 10×, and 43.79% at 20×, indicating that the vast majority of reads were successfully mapped to the reference genome with high-quality alignments. A total of 29.54 Tb of raw sequencing data was obtained. The GC content of the 474 samples ranged from 41.98% to 47.05%, with an average of 44.20%. After quality control, the average Q20 and Q30 scores were 98.91% and 97.28%, respectively. These results demonstrate reliable sequencing quality and no obvious bias during library preparation or sequencing. To ensure the reliability of subsequent analyses, stringent quality control was conducted using PLINK (v.1.90) [30]. Variants and individuals were filtered according to the following criteria: variant call rate < 90% (--geno 0.1), Hardy–Weinberg equilibrium *p* value < 10^−6^ (--hwe 1e-6), minor allele frequency (MAF) < 0.01 (--maf 0.01), and individual call rate < 90% (--mind 0.1).

### 2.3. Population Structure and Linkage Disequilibrium Analysis

To reduce false positives caused by population stratification in GWAS, principal component analysis (PCA) was performed using the rMVP package (v1.4.6) [31]. The top three principal components (PC1–PC3) were extracted and incorporated as covariates in subsequent association models. To evaluate pairwise genetic relatedness among individuals, a genomic relationship matrix (G-matrix) was constructed with GCTA and included in the analytical models [32]. In addition, PopLDdecay (v.3.4.0) was used to calculate the genome-wide linkage disequilibrium (LD, measured by r^2^) decay distance, which was used to define the search window for candidate genes [33].

### 2.4. Multi-Model Genome-Wide Association Study (GWAS)

To accurately identify genetic variants significantly associated with ADG and effectively control false-positive and false-negative rates, three distinct statistical models—General Linear Model (GLM), Mixed Linear Model (MLM), and Fixed and Random Model Circulating Probability Unification (FarmCPU)—were jointly implemented using the rMVP package in R (v4.5.1). Systematic non-genetic environmental factors were incorporated into the statistical models: sex (two levels: male and female) and measurement batch (six levels based on rearing areas/sub-farms) were treated as categorical fixed effects, while birth weight (continuous variable) and the top three principal components (PC1–PC3, continuous variables) were included as quantitative covariates to correct for population structure and individual body size differences. For individual missing values in phenotypic records, they were uniformly coded as NA in the input files, and the complete cohort of 474 genotyped individuals was retained for analysis. To alleviate the stringent multiple testing penalty caused by the massive marker density in whole-genome resequencing (WGS) data and improve the iterative computational efficiency of mixed models such as FarmCPU, genome-wide high-quality SNPs were subjected to linkage disequilibrium (LD) pruning using PLINK v1.90 with a sliding window of 50 SNPs, a step size of 5 SNPs, and a pairwise r^2^ threshold of 0.2. Given our high-density WGS dataset, a window of 50 SNPs corresponds to an average physical genomic window of approximately 5.5–6.0 kb. After pruning, a total of 3,358,628 independent representative SNPs were retained for association analysis. A strict Bonferroni correction was applied to determine the significance threshold, defined as the chromosome-wide threshold *p* = 1/3,358,628. For clear visualization in Manhattan plots, this threshold was converted to the −log_10_(*p*) scale. Manhattan and quantile-quantile (Q-Q) plots were generated using the rMVP package.

### 2.5. Estimation of Genetic Parameters and Genomic Selection (GS)

Prior to GBLUP modeling, 27 individuals were excluded from the initial cohort of 474 Ashidan yaks due to incomplete 6-month body weight records or missing ADG phenotypes, leaving a final analytical set of 447 qualified animals with complete phenotypic and genotypic profiles to maintain high phenotypic quality and model stability. Unlike the LD pruning scheme adopted for GWAS, we retained the full set of high-density variants to construct genomic selection models. The univariate genomic best linear unbiased prediction (GBLUP) algorithm implemented in HIBLUP v.1.6.0 was used to decompose variance components and calculate genomic estimated breeding values (GEBVs). The mixed linear model is specified as follows:

y=Xb+Zu+e

where *y* is the vector of 6-month ADG phenotypic values; *b* is the vector of fixed environmental effects and continuous covariates, including sex (2 levels: male/female), measurement batch (6 levels based on sub-farm rearing areas), birth weight, and PC1–PC3, consistent with the GWAS models; u is the vector of additive genetic effects (GEBVs) following a normal distribution $N(0,G/\sigma_{g}^{2})$, in which G is the genomic relationship matrix constructed using all quality-controlled SNPs. *X* and *Z* are the design matrices corresponding to b and u, respectively, and e is the vector of random residuals. The formula for calculating the narrow-sense heritability (*h*^2^) of ADG is presented as follows:

$$h^{2}=\frac{\sigma_{a}^{2}}{\sigma_{a}^{2}+\sigma_{e}^{2}}$$

To evaluate the predictive performance of the GBLUP model, a random 10-fold cross-validation scheme was implemented with a fixed random seed. The 447 qualified animals were randomly partitioned into ten roughly equal subsets. In each iteration, nine subsets served as the reference population for model training, while the remaining one subset acted as the validation population with masked phenotypes. Prediction accuracy was defined as the Pearson correlation coefficient (R) between the GEBVs predicted by the model and the adjusted phenotypic values. Furthermore, the additive effect weights of all SNPs were extracted using the --snp-effect parameter in HIBLUP [34]. This step aimed to screen high-weight loci and provide critical target markers for the future development of a custom breeding chip for Ashidan yaks. To eliminate potential data leakage and ensure an unbiased genomic evaluation, SNP prioritization was conducted strictly and independently within each training fold during the 10-fold cross-validation. In each of the ten iterations, marker additive effect sizes were calculated exclusively using the reference cohort to extract the fold-specific top 1% SNPs (~228,000 loci). These fold-specific core markers were then used to construct the genomic relationship matrix for predicting GEBVs in the completely masked validation cohort (1/10 of animals).

### 2.6. Gene Annotation and GO/KEGG Enrichment Analysis

Based on the linkage disequilibrium (LD) decay distance of the Ashidan yak population, the Ensembl database (LU_Bosgru_v3.0) was used to identify positional candidate genes located in the upstream and downstream flanking regions of genome-wide significant SNPs. To further elucidate the biological functions and metabolic pathways of these candidate genes, Gene Ontology (GO) functional annotation and Kyoto Encyclopedia of Genes and Genomes (KEGG) pathway enrichment analyses were performed. Multiple-testing correction was applied using the Benjamini–Hochberg false discovery rate (FDR) procedure to control for false positives. Functional terms and signaling pathways with *p* < 0.05 and FDR-adjusted *p*-values (FDR < 0.05 or Q < 0.10) were considered statistically significantly enriched. Both raw *p*-values and adjusted FDR values (*p*.adjust) are comprehensively reported in the manuscript and Supplementary Table S2 for complete statistical transparency.

## 3. Results

### 3.1. Descriptive Statistics of Phenotypes, Variant Quality Control and Population Structure Analysis

**Figure 1:**
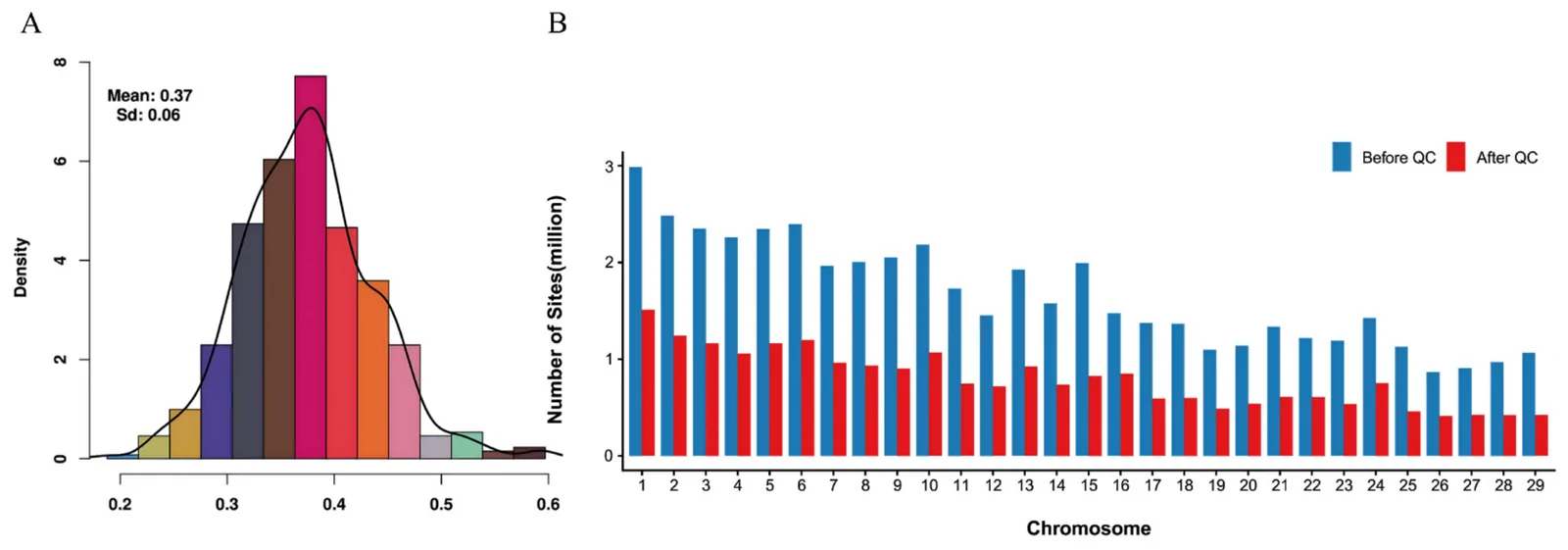
Phenotypic distribution of average daily gain (ADG) (**A**) and quality control results of genome-wide variants (**B**) in the Ashidan yak population. (**A**) The mean ADG was 0.37 kg/day with a standard deviation of 0.06. The phenotypic data followed a normal distribution, which was suitable for subsequent quantitative genetic analyses. (**B**) Comparison of variant numbers across 29 autosomes before and after quality control (QC). Blue bars represent raw variants obtained from resequencing (Before QC), and red bars indicate high-quality SNPs retained after stringent filtering (After QC).

High-depth whole-genome resequencing data were obtained from a total of 474 Ashidan yaks. For the phenotypic trait of average daily gain (ADG), the mean value was 0.375 kg/day with a standard deviation of 0.061 and a coefficient of variation of 16.329%. The results revealed abundant phenotypic variation in ADG within the experimental population, indicating great potential for genetic selection (Figure 1A).

A large number of raw genetic variants were initially detected across 29 autosomes during variant calling and quality control (QC). To guarantee the reliability of high-resolution genome-wide association study (GWAS) and genomic selection (GS), strict filtering criteria were applied: variants with genotype missing rate > 10% (--geno 0.1), Hardy–Weinberg equilibrium *p* value < 10^−6^ (--hwe 1 × 10^−6^), minor allele frequency (MAF) < 0.01 (--maf 0.01), and individuals with missing rate > 10% (--mind 0.1) were removed. As shown in Figure 1B, variant numbers on each autosome decreased uniformly after QC, effectively removing low-quality spurious variants. Finally, a total of 22,870,535 high-quality SNPs were retained. The distribution of these SNPs across chromosomes was positively correlated with chromosomal physical length, providing a robust dataset for subsequent genetic analyses.

**Figure 2:**
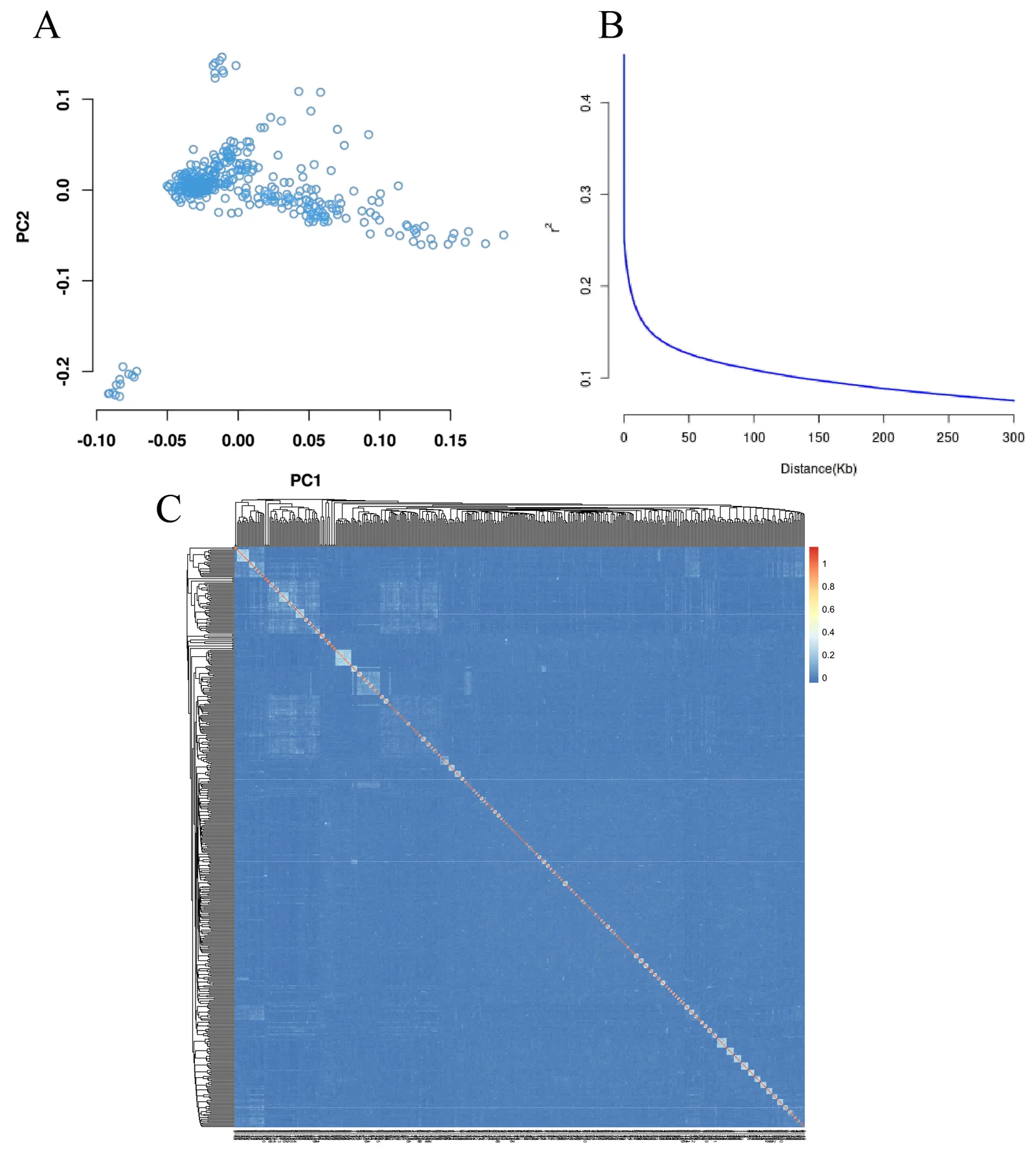
Population structure, linkage disequilibrium (LD) decay, and genomic relationship analysis of the Ashidan yak population. (**A**) Principal component analysis (PCA) plot of PC1 versus PC2, showing a relatively continuous and homogeneous genetic structure. (**B**) Genome-wide average LD decay (r^2^) over physical distance (kb), reaching r^2^ = 0.1 at approximately 128.28 kb. (**C**) Heatmap of the genomic relationship matrix (G-matrix) constructed from high-density SNPs among 474 Ashidan yaks. The color gradient from blue to red represents pairwise genetic coefficients (0 to 1), indicating that the vast majority of individuals exhibit distant/weak genetic relatedness, with strong relationships restricted strictly to sparse diagonal clusters.

Principal component analysis (PCA) based on high-quality genome-wide SNPs was performed on all 474 sequenced individuals to control false positives in GWAS. The scatter plot of the first two principal components (PC1 and PC2) showed that most individuals clustered closely without obvious population stratification or extensive genetic divergence, suggesting a relatively homogeneous genetic structure of this selectively bred yak population (Figure 2A). Genome-wide linkage disequilibrium (LD) decay analysis revealed that the physical distance was approximately 128.28 kb when the r^2^ value declined to 0.1. This LD decay distance reflected the evolutionary and artificial selection history of Ashidan yaks (Figure 2B), and was defined as the search window for screening candidate genes surrounding significant GWAS loci (Figure 2C).

### 3.2. Identification of Core Markers and Candidate Genes for ADG via Multi-Model Genome-Wide Association Study (GWAS)

**Figure 3:**
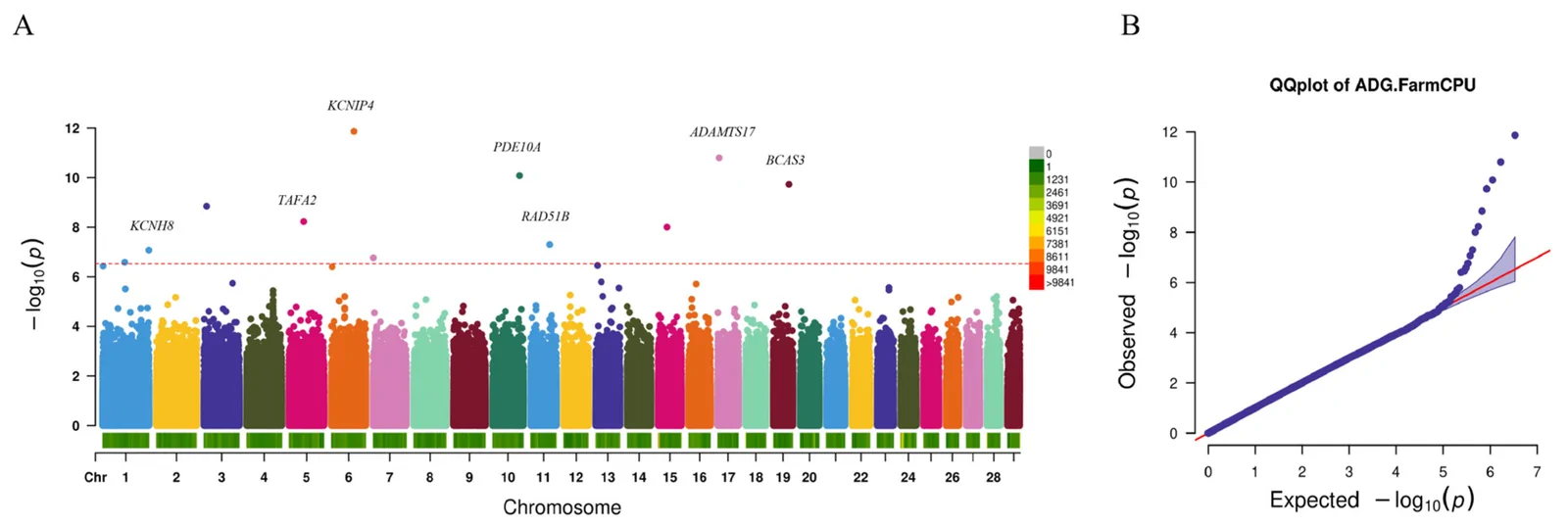
Manhattan plot (**A**) and Q-Q plot (**B**) of average daily gain (ADG) traits in Ashidan yak. The red solid line indicates the genome-wide Bonferroni significant threshold (−log_10_(*p*) = 6.53). Points exceeding the red line represent genome-wide significantly associated SNP loci. The Q-Q plot compares observed −log_10_(*p*) values against expected −log_10_(*p*) values, with a genomic inflation factor λ = 1.0278 demonstrating effective correction of population stratification; the red diagonal line corresponds to the null distribution of P-values under no association.

To explore the molecular genetic basis of average daily gain (ADG) in Ashidan yaks, genome-wide association analysis was performed on 474 individuals using 3,358,628 high-resolution independent SNPs obtained via genome-wide linkage disequilibrium (LD) pruning. Three models, namely the General Linear Model (GLM), Mixed Linear Model (MLM) and FarmCPU, were applied in parallel for cross-validation and performance evaluation. Genomic inflation factor (lambda) and quantile-quantile (Q-Q) plots revealed that the observed *p*-values of GLM deviated substantially from the expected null distribution due to uncorrected cryptic population structure and individual relatedness, resulting in severe genomic inflation (Supplementary Figure S1). In contrast, the lambda values of MLM and FarmCPU were close to 1.0, and the observed trajectories in Q-Q plots closely matched the expected diagonal line (Figure 3, Supplementary Figure S2), demonstrating that mixed models effectively eliminated systematic false positives caused by population stratification. Comparative analysis of model performance indicated that FarmCPU achieved higher statistical power while rigorously controlling spurious associations. Given the severe multiple testing penalty derived from dense whole-genome resequencing markers, the genome-wide significance threshold was *p* = 1/3,358,628 based on the number of independent pruned SNPs, corresponding to −log_10_ (*p*) = 6.53 for Manhattan plot visualization. Under this strict threshold, a total of 11 genome-wide significant independent variants associated with ADG were identified using FarmCPU (Figure 3A).

Using the previously determined LD decay distance of 128.28 kb as the search window, the physical positions of significant loci were mapped to the yak reference genome (LU_Bosgru_v3.0). Seven functional candidate genes were annotated in the flanking regions, including *KCNH8*, *TAFA2*, *KCNIP4*, *PDE10A*, *RAD51B*, *ADAMTS17* and *BCAS3*. These genes and their tightly linked variant regions constitute the quantitative trait loci (QTLs) underlying early growth and daily gain in Ashidan yaks.

### 3.3. Functional Annotation and Pathway Enrichment Analysis of Candidate Genes

**Figure 4:**
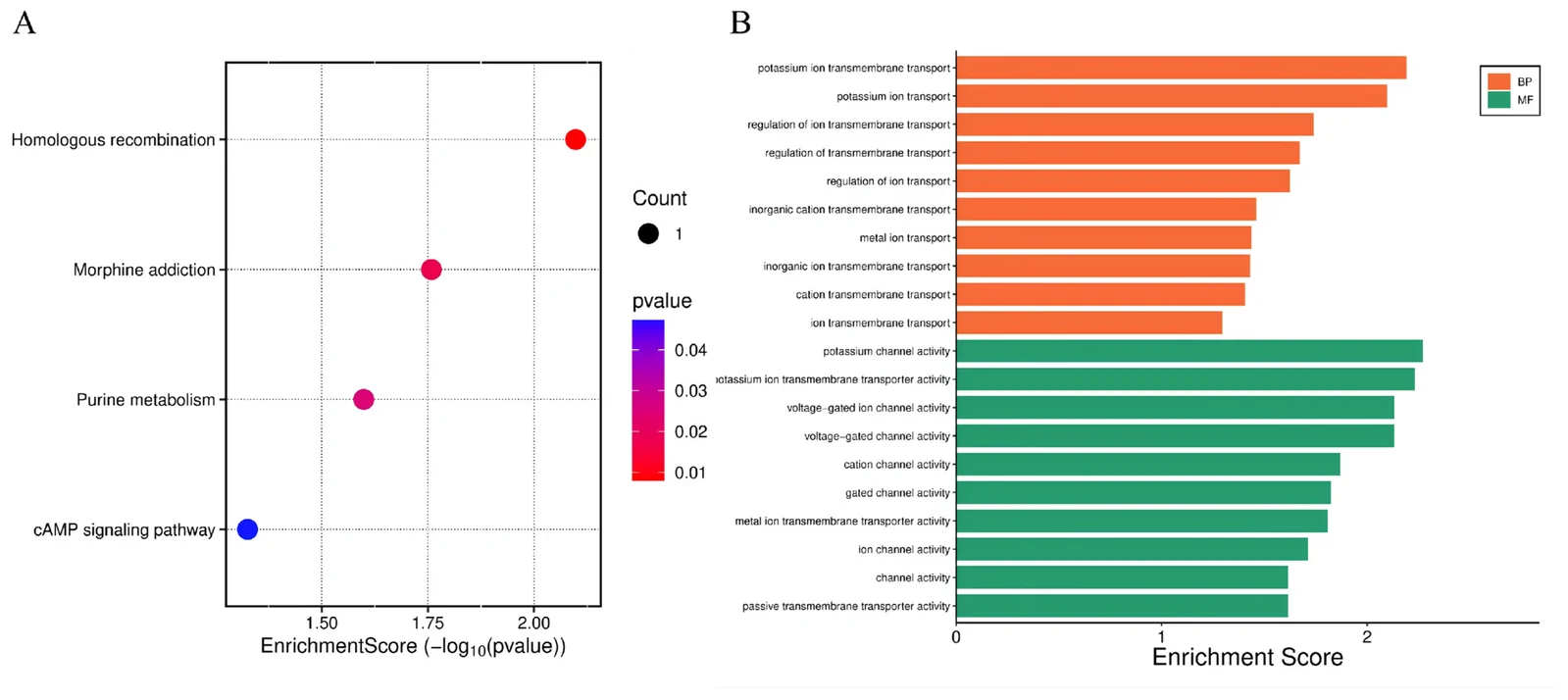
KEGG pathway enrichment (**A**) and GO functional enrichment analysis (**B**) of candidate genes significantly associated with early average daily gain in Ashidan yaks.

Based on the 11 genome-wide significant SNPs detected by FarmCPU and the 128.28 kb LD decay window, seven positional candidate genes were annotated within the associated regions. To clarify their potential biological roles in the growth and development of Ashidan yaks, Kyoto Encyclopedia of Genes and Genomes (KEGG) enrichment analyses (Figure 4A) and Gene Ontology (GO) (Figure 4B) were performed, with multiple-testing corrections applied using the Benjamini–Hochberg false discovery rate (FDR) procedure.

GO enrichment analysis revealed functional profiles across Biological Process (BP), Cellular Component (CC), and Molecular Function (MF) categories. Candidate genes were significantly enriched in ion channel and transport processes, including potassium channel activity (*p* = 0.0054, FDR = 0.0315), potassium ion transmembrane transport (*p* = 0.0064, FDR = 0.0560), and voltage-gated ion channel activity (*p* = 0.0074, FDR = 0.0315). These findings suggest that candidate genes regulate energy metabolism and cellular homeostasis by modulating transmembrane ion flux and excitability in muscle and nervous tissues. KEGG pathway analysis further mapped the candidate genes into core metabolic and signaling networks. Following Benjamini–Hochberg correction, all top enriched pathways retained statistical significance (FDR < 0.05), including Homologous recombination (*p* = 0.0080, FDR = 0.0319), Morphine addiction (*p* = 0.0174, FDR = 0.0336), Purine metabolism (*p* = 0.0252, FDR = 0.0336), and the cAMP signaling pathway (*p* = 0.0472, FDR = 0.0472). Notably, purine metabolism and the cAMP signaling pathway serve as central regulatory hubs for energy sensing and cellular signal transduction, which play essential roles in muscle fiber development and growth hormone regulation. Detailed statistics for all GO terms and KEGG pathways, including raw *p*-values and Benjamini–Hochberg adjusted FDR values (p.adjust), are comprehensively presented in Supplementary Table S2.

### 3.4. Partitioning of Additive Genetic Variance and Evaluation of Genomic Selection

To systematically assess the genomic breeding value of average daily gain (ADG) and lay quantitative foundations for molecular genetic improvement of Ashidan yaks, we conducted genetic parameter estimation and genomic selection (GS) analyses following the GWAS-based dissection of ADG regulatory architecture. Distinct from the linkage disequilibrium pruning strategy used in GWAS to reduce marker dimensionality, we retained the full panel of 22,870,535 high-quality genome-wide SNPs to comprehensively capture minor polygenic additive effects. Prior to GS modeling, 27 animals lacking complete six-month ADG phenotypic data were removed from the cohort, yielding a final analytical set of 447 qualified individuals. Using this filtered population, we constructed a genomic relationship matrix (G-matrix) with HIBLUP and decomposed additive genetic variance via the univariate genomic best linear unbiased prediction (GBLUP) model.

Variance component estimates (Table 1) showed that the genomic additive genetic variance (V_a_) and residual variance (V_e_) of ADG were 0.00104 ± 0.00054 and 0.00218 ± 0.00052, respectively. The estimated narrow-sense genomic heritability (h^2^) was 0.3233 ± 0.1633, which was statistically significant (likelihood ratio test, *p* = 0.0478 < 0.05). The results indicated that ADG exhibited moderate to high heritability in this population. Collectively, ADG is substantially controlled by polygenic additive variants, which lays a solid theoretical foundation for early genomic selection in Ashidan yak breeding.

**Figure 5:**
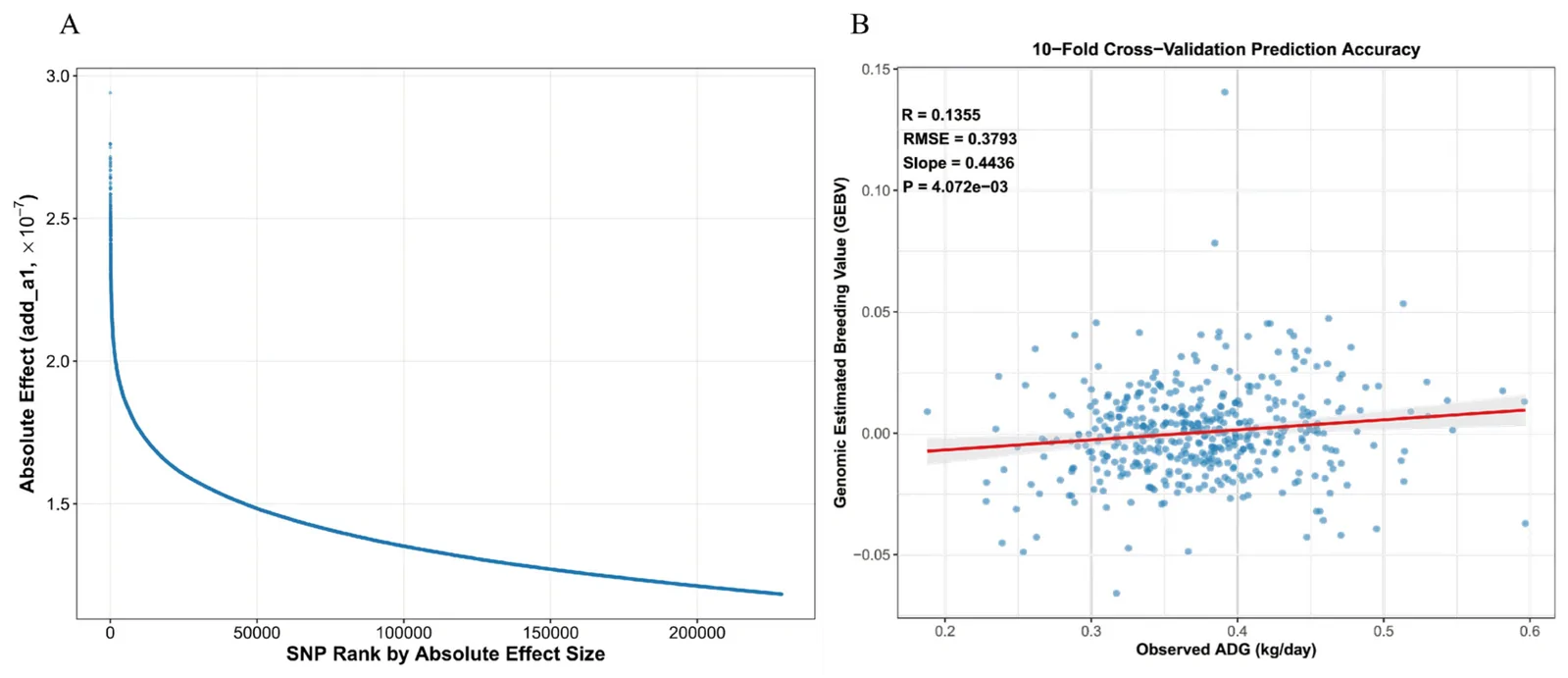
Genome-wide marker effect distribution and prediction accuracy for average daily gain (ADG) in Ashidan yaks. (**A**) Distribution of absolute additive effects of genome-wide SNPs. Most genetic variation is governed by a small number of high-impact loci, showing an exponential decay pattern. The figure also illustrates the distribution of the top 1% SNPs (approximately 228,000 loci) selected as core targets for the breeding array. (**B**) Scatter plot of adjusted observed ADG versus genomic estimated breeding values (GEBVs) for individuals in the validation set from 10-fold cross-validation. The red line represents the linear regression trend, and the shaded area indicates the 95% confidence interval. The Pearson correlation coefficient (R) and evaluation metrics are presented in the upper-left corner.

To further dissect the genetic architecture of average daily gain (ADG), we extracted the predicted additive effects of all 22.87 million genome-wide SNPs from the primary GBLUP model (Figure 5A). The visualization presented a typical L-shaped exponential decay pattern consistent with the polygenic inheritance model: a tiny fraction of top-ranked variants captured a substantial portion of the additive genetic variance, while the vast majority of background markers exerted negligible effects. Under the full 22.87-million SNP dataset, the 10-fold cross-validation scheme yielded an average independent prediction accuracy of R = 0.14 ± 0.07 (with the overall pooled scatter plot and evaluation metrics across the 10-fold validation presented in Figure 5B). These baseline results demonstrate that early individual selection using high-density WGS data is statistically feasible.

To evaluate whether filtering out background noise could enhance prediction efficiency, we prioritized the top 1% SNPs (approximately 228,000 loci) with the largest absolute additive effect sizes to construct a reduced core marker panel. Notably, these core markers showed substantial physical overlap with candidate genes and biological pathways identified via GWAS. Under the strict 10-fold within-fold cross-validation scheme, the unbiased prediction accuracy using the fold-specific top 1% SNP panel averaged R = 0.1328 ± 0.1566, with an average RMSE of 0.3793 ± 0.0066 and an average regression slope of 0.4183 ± 0.5055. Compared with the unselected whole-genome baseline (R = 0.14 ± 0.07), prioritizing markers solely based on single-trait GBLUP effect magnitudes within small training folds did not yield an accuracy boost. This demonstrates that marker effect estimations in modest reference populations are subject to sampling variance when evaluated under strict, leak-free cross-validation.

## 4. Discussion

### 4.1. Multi-Model GWAS for ADG and Functional Analysis of Key Candidate Genes

Average daily gain (ADG) is a crucial quantitative trait that determines economic benefits and large-scale breeding potential of meat-type yaks [35,36]. The present study revealed that Ashidan yaks maintained considerable phenotypic variation in ADG (coefficient of variation, CV = 16.329%) under the harsh alpine rearing conditions of the Qinghai–Tibet Plateau. Such moderate phenotypic variation reflects the complex interactions between genetic potential and extreme environments, offering opportunities for genetic evaluation and molecular selection in this population.

Based on high-depth whole-genome resequencing (WGS) of 474 individuals and stringent quality control, we constructed an ultra-high-density variation map containing more than 22.87 million high-quality SNPs. This dataset increased genomic coverage, enabling high-resolution mapping of candidate regions associated with ADG.

In conventional GWAS, potential population stratification and intricate kinship derived from long-term artificial selection within the Ashidan yak population tend to generate numerous false-positive signals. To address this issue, we performed strict linkage disequilibrium (LD) pruning specifically for GWAS to eliminate redundant linkage background noise. Furthermore, a parallel comparison of multiple association models clearly highlighted their distinct statistical performance in population stratification control. The single-marker General Linear Model (GLM) produced marked genomic inflation (λ = 1.1077) because it failed to account for cryptic relatedness and complex family structures among individuals, leading to elevated false-positive associations. In contrast, the Mixed Linear Model (MLM) effectively eliminated these systematic false positives (λ = 1.0089) by incorporating the genomic relationship matrix (G-matrix) as a random effect to capture genome-wide background relatedness. To further overcome the statistical limitation of conventional MLM—where candidate variants included within the G-matrix suffer from over-correction and high false-negative rates—we implemented the FarmCPU algorithm. By iteratively fitting pseudo-QTNs as fixed-effect covariates while retaining a restricted kinship matrix in the random model, FarmCPU completely decouples fixed and random effects, achieving ideal genomic inflation control (λ = 1.0278). Consequently, FarmCPU achieved superior statistical power in uncovering genuine quantitative trait loci (QTLs) for average daily gain while rigorously guarding against both false positives and false negatives, ultimately identifying 11 high-confidence SNPs and seven core candidate genes.

Four candidate genes, *PDE10A*, *TAFA2*, *KCNH8* and *KCNIP4*, were found to function in the neuroendocrine axis and the regulation of metabolic efficiency. As a vital member of the phosphodiesterase family, *PDE10A* is indispensable for intracellular cyclic nucleotide (cAMP/cGMP) signaling, and it directly mediates hypothalamic regulation of body temperature and energy homeostasis. Nawrocki et al. demonstrated that *PDE10A*-deficient mice are resistant to diet-induced obesity and related metabolic disorders [37]. This resistance stems from hypophagia when consuming a high-fat, high-sugar palatable diet, but not a standard chow diet. *PDE10A* deficiency reduces caloric intake without altering meal frequency, diurnal feeding patterns, or locomotor activity. In yaks, genetic variations in *PDE10A* may optimize basal metabolic rate and feeding motivation under harsh plateau conditions by modulating hypothalamic cAMP signaling, thereby potentially influencing ADG traits. A GWAS conducted by Martínez-Montes et al. across three pig populations with distinct genetic backgrounds confirmed that *PDE10A* is a pivotal gene underlying quantitative trait loci (QTLs) for body weight. *PDE10A* identified in Ashidan yaks serves as a potential candidate gene associated with growth traits [38]. Additionally, *TAFA2* (also known as *FAM19A2*), a neurochemokine, has been reported to participate in appetite regulation and metabolic feedback in the central nervous system of animals [39]. Meanwhile, both *KCNH8* and KCNIP4 encode key voltage-gated potassium and calcium channel proteins expressed in the central nervous system and muscle tissues. Recent GWAS on beef cattle have repeatedly verified that these two genes are significantly associated with dry matter intake (DMI) and feed conversion efficiency [40,41]. For Ashidan yaks chronically exposed to cold stress on the Qinghai–Tibet Plateau, this gene cluster dominated by ion channels and the cAMP signaling pathway likely enhances basal metabolic rate and improves feeding feedback under plateau conditions, thereby sustaining their growth potential by regulating energy metabolism.

The genes *RAD51B*, *ADAMTS17* and *BCAS3* play fundamental roles in regulating skeletal muscle growth and body size development. Previous GWAS on commercial beef cattle have confirmed that RAD51B is strongly associated with carcass tissue development and body size traits in Nellore cattle [42]. *ADAMTS17* belongs to the extracellular matrix (ECM) metalloproteinase family. In a study by Yu et al. GWAS identified eight candidate genes including *ADAMTS17*, *ALDH1A3*, *CHSY1*, *MAGEL2*, *MEF2A*, *SYNM*, *CNTNAP5* and *CTNNA3*, which are most likely involved in regulating growth traits of Qinchuan cattle [19]. A review by Stylianos Z. Karoulias noted that mutations in *ADAMTS10* and *ADAMTS17* cause Weill–Marchesani syndrome (WMS), a disorder mainly affecting the musculoskeletal system and leading to short stature, lens dislocation and cardiac valve abnormalities [43]. Furthermore, *BCAS3* has been well documented to regulate angiogenesis and cytoskeleton remodeling, which establishes the microvascular network required for rapid proliferation of muscle tissues. Molecular cytological studies have verified that *BCAS3* encodes a highly conserved cytoskeleton-regulatory protein, which acts as an essential regulator mediating directional migration and sprouting angiogenesis of endothelial cells [44]. During the early exponential growth stage of mammals, the rapid hypertrophy of skeletal muscle fibers relies heavily on the synchronous expansion of intramuscular microvascular networks to meet high demands for oxygen and nutrient metabolism. For Ashidan yaks living chronically in the extreme cold and hypoxic environment of the Qinghai–Tibet Plateau, *BCAS3* is hypothesized to participate in vascularization processes during skeletal muscle development, though functional validation is required. A GWAS on milk yield in Holstein cows conducted by Esrafili et al. identified genes including *BCAS3*, *MALRD1* and *CTNND2*. This further confirms the vital role of *BCAS3* in regulating intensive nutrient metabolism and productive performance in bovines [45]. Whole-genome resequencing and GWAS for body length and body weight in Cynoglossus semilaevis conducted by Wang et al. also demonstrated associations between *BCAS3* and these growth traits, along with *AKAP9* and *ANAPC2* [46]. We therefore speculate that polymorphisms of *BCAS3* identified in Ashidan yaks may facilitate three-dimensional growth of skeletal muscle by promoting microvascular sprouting and nutrient transport, ultimately contributing to the economically important high average daily gain.

To further verify the combined regulatory mechanisms of these candidate genes at the systems biology level, we performed GO functional annotation and KEGG pathway enrichment analysis on the significantly associated target genes. The results revealed that these polymorphic loci do not function independently, but are predominantly enriched in core pathways related to energy metabolic homeostasis, extracellular matrix remodeling and transmembrane ion transport.

In terms of KEGG pathway enrichment, the candidate genes were significantly clustered in the cAMP signaling pathway as well as calcium and potassium ion signal transduction pathways. As mentioned above, *PDE10A*, a key enzyme modulating cAMP levels, may exert potential functional synergy with *KCNH8* and *KCNIP4*, which are enriched in ion channel pathways. A study by Sharma et al. revealed that the melanocortin 4 receptor (*MC4R*) is a key regulator of satiety and feeding behavior, and its mutations represent the most common cause of monogenic obesity. *MC4R* transduces signals via multiple G protein subtypes. Specifically, the Gαq-coupled pathway in the paraventricular nucleus is essential for normal satiety, and this pathway triggers intracellular Ca^2+^ release [47]. Furthermore, ghrelin, an orexigenic hormone, activates its receptor GHSR1a to enhance the phosphorylation of hippocampal NMDA receptors. This process relies on G protein-regulated second messengers, including cAMP and calcium signaling [48]. In addition to the aforementioned G protein subtypes, Gαs, Gαi/o and Gα12/13 are also involved in MC4R-mediated signal transduction. Gαs is a canonical subtype that activates adenylate cyclase and elevates intracellular cAMP levels. By contrast, Gαi/o suppresses adenylate cyclase activity, thereby decreasing cAMP synthesis. Gα12/13 modulates cytoskeletal rearrangement through the RhoA pathway. cAMP and Ca^2+^ signals not only rapidly regulate neuronal excitability, but also mediate long-term appetite regulation by modulating gene expression. A previous study on ducks revealed distinct hypothalamic gene expression profiles between individuals with low and high residual feed intake (RFI). Animals with low RFI are characterized by high feed efficiency, and their differentially expressed genes were significantly enriched in the cAMP signaling pathway and calcium signaling pathway [49,50]. Given that cAMP and calcium signaling are universally involved in regulating the excitability of hypothalamic appetite neurons and energy metabolism in skeletal muscle across mammals, the prominent enrichment of these pathways implies that the enhanced average daily gain (ADG) of Ashidan yaks is partially driven by genetic improvements in energy conversion efficiency and central feeding motivation. In the GO enrichment analysis for Cellular Component and Biological Process, multiple significantly enriched terms were associated with extracellular matrix organization, angiogenesis and cytoskeletal protein binding. These results are highly consistent with the known physiological functions of the candidate genes identified herein: *ADAMTS17* regulates the extracellular matrix and microfibril network, whereas *BCAS3* modulates cytoskeleton dynamics and microvascular sprouting. The coordinated expansion of the extracellular matrix and microvascular network is widely recognized as an essential physiological basis for skeletal muscle hypertrophy and three-dimensional body growth in mammals. Accordingly, systematic GO and KEGG analyses not only further validate the GWAS results, but also offer potential clues to decipher the biological network governing ADG in Ashidan yaks. In this population, the cAMP/ion channel pathways are hypothesized to facilitate energy intake and conversion, while extracellular matrix and angiogenesis pathways likely offer spatial and microenvironmental support during muscle development.

### 4.2. Estimation of Genetic Parameters and Genomic Prediction Efficiency for Average Daily Gain in Ashidan Yaks

Although the above GWAS and enrichment analyses have preliminarily characterized the major gene network regulating average daily gain (ADG), this complex quantitative trait is generally governed by a small number of major loci combined with numerous polygenic variants of minor effect. Conventional GWAS inevitably misses a large proportion of minor-effect variants due to the strict statistical significance thresholds and linkage disequilibrium (LD) pruning for dimensionality reduction and false positive control, which consequently leads to the well-known issue of missing heritability [51]. To comprehensively evaluate the overall breeding potential of the Ashidan yak population, we adopted a strategy of retaining all genetic markers for genomic selection (GS), which differs from that used in GWAS. A total of 22,870,535 high-density SNPs were directly incorporated into the GBLUP model.

Variance component decomposition showed that the constructed unbiased genomic relationship matrix (G-matrix) effectively captured the underlying additive genetic variance of the population. The narrow-sense genomic heritability (h^2^) of ADG in Ashidan yaks was estimated to be 0.3233 (*p* < 0.05). According to classical quantitative genetics, the estimated narrow-sense genomic heritability (h^2^ = 0.3233, *p* < 0.05) represents a moderate-to-high magnitude for bovine growth traits. This indicates that despite extreme alpine environmental stress, approximately 32% of the total phenotypic variance in early average daily gain can be attributed to polygenic additive genetic factors. Quantitative traits with moderate-to-high heritability generally respond rapidly and favorably to directional selection.

In practical terms, this moderate heritability offers a useful quantitative baseline for early genomic evaluation. Coupled with the genomic prediction framework established in this study, breeders can evaluate genomic estimated breeding values (GEBVs) in young calves right after birth or weaning. This eliminates the long wait for animals to reach adult slaughter age under harsh grazing conditions, thereby substantially shortening generation intervals and accelerating genetic progress. Nevertheless, because quantitative traits are heavily influenced by non-genetic environmental factors (such as pasture nutrition, micro-climate, and management), incorporating crucial fixed effects (e.g., sex, age, and rearing sub-farm batches) into genomic evaluation models is imperative to avoid systematic bias in breeding values. This genomic heritability estimate is consistent with reported ranges for growth traits in beef cattle [52,53,54], providing a useful quantitative reference for future genetic evaluation in Ashidan yaks. Under the full high-density marker dataset, 10-fold cross-validation yielded a modest baseline prediction accuracy (R = 0.14 ± 0.07). This baseline result aligns with reported prediction accuracies (ranging from 0.17 to 0.296) for growth traits in Chinese commercial beef cattle populations using unselected markers in GBLUP models [55].

In practical applications of genomic selection, genetic evaluation for indigenous livestock is often constrained by the high cost of phenotyping and the challenge of establishing large reference populations [45,56]. Given the extreme environmental conditions on the Qinghai–Tibet Plateau and the modest sequencing sample size in this study, this baseline accuracy reflects the inherent difficulty of predicting polygenic traits using unselected whole-genome markers. Rather than supporting immediate large-scale field selection, these findings provide a valuable quantitative baseline and preliminary insights. They emphasize that optimizing marker sets and controlling non-genetic noise are essential steps before genomic estimated breeding values (GEBVs) can be effectively integrated into routine early selection for young yaks.

### 4.3. Analysis of Polygenic Effect Distribution for ADG and Prospect of Future Genomic Breeding Strategies

To further elucidate the underlying genetic architecture of ADG in Ashidan yaks from a global perspective, we extracted the predicted additive effects of more than 22.87 million genome-wide variants from the GBLUP model. Visualization results revealed a distinct L-shaped exponential decay pattern for the effects of genome-wide markers. This quantitative genetic phenomenon demonstrates that ADG is regulated by numerous minor-effect polygenes. A tiny fraction of top-ranked variants with large effects constitute the core framework of additive genetic variance for this trait, while the vast majority of background variants only exert minor polygenic modulatory effects. This finding validates the finite polygenic model for complex traits in the Ashidan yak population, and also explains why a moderately high narrow-sense genomic heritability (h^2^ = 0.3233) was accurately estimated despite interference from the extreme alpine environment. The choice of the top 1% SNP threshold (approximately 228,000 loci) for core marker prioritization was strictly justified by the observed L-shaped exponential decay pattern of marker additive effects (Figure 5A). Quantitatively, this 1% threshold captured the primary proportion of additive genetic variance while effectively filtering out millions of uninformative background variants that introduce genomic relationship estimation noise. Although alternative tighter candidate thresholds (e.g., top 0.1% or 0.5%) were evaluated during exploratory screening, they primarily captured genome-wide significant GWAS loci but omitted a considerable fraction of polygenic minor-effect variants, leading to reduced total variance capture. Crucially, our re-evaluation under strict within-fold cross-validation revealed an unbiased prediction accuracy of R = 0.0991 ± 0.0697. Comparing this leak-free result with the unselected baseline (R = 0.14 ± 0.07) illustrates that in small reference populations (n = 447), individual marker effect estimations via GBLUP suffer from substantial sampling noise. Prioritizing loci solely by effect magnitude within small training subsets cannot reliably isolate true polygenic signals. Therefore, for practical low-cost custom array design in indigenous yaks, marker selection should not rely purely on single-trait effect sorting, but should strategically incorporate GWAS peak loci, SnpEff functional variant annotations, and biological pathway information.

From the perspective of industrial application in modern animal breeding, whole-genome deep resequencing (WGS) provides high-resolution variant detection, yet its high genotyping cost and intensive computational demands hinder its large-scale direct application in pastoral areas and core breeding farms. To address this challenge, we performed strict screening by sorting variants in descending order of effect size, and retained the top 1% variants with the largest absolute effects, approximately 228,000 loci in total (see Supplementary Table S1). These loci serve as valuable candidate targets for developing a low-cost custom breeding array tailored for early selection of meat production performance in Ashidan yaks in cooperation with array manufacturing platforms. For practical industrial deployment, designing a low-cost, customized liquid or solid-phase SNP array is pivotal to bridging whole-genome sequencing with routine field selection in yaks. Based on the genome-wide LD decay distance (approx. 128 kb) and our empirical cross-validation results, we recommend a low-to-medium density panel comprising approximately 10,000 to 50,000 prioritized SNPs (~10K–50K chip). To optimize array content and minimize manufacturing costs, redundant markers in strong linkage disequilibrium (r^2^ > 0.2) should be strictly filtered out via window-based pairwise LD pruning, ensuring that selected tag-SNPs are evenly distributed across chromosomes to maximize genotype imputation efficiency. Furthermore, long-term genomic selection inevitably drives rapid shifts in allele frequencies, where directional selection for ADG may alter frequencies at pleiotropic loci and induce unintended negative genetic correlations with other vital economic traits. To mitigate these adverse correlated responses, future customized arrays should integrate multi-trait GWAS targets and functional variants. Moreover, genomic selection strategies should incorporate multi-trait selection indices or constrained GBLUP models to balance weight gain with maternal and health traits, thereby preserving population genetic diversity while sustaining long-term genetic gain.

Based on these findings, such compact and targeted genotyping tools can be adopted in future breeding practices to calculate genomic estimated breeding values (GEBVs) and select candidates with high ADG potential at an early age. This approach reduces reliance on long-term field phenotyping under harsh alpine conditions and may shorten generation intervals, offering practical insights for low-cost genomic evaluation of indigenous livestock on the Qinghai–Tibet Plateau.

A balanced interpretation of our findings requires acknowledging several study limitations. First, the candidate genes identified via GWAS represent statistical associations rather than direct functional causality; precise regulatory mechanisms and potential causal variants require future verification through functional genomic assays and multi-omics integration. Second, the reference population size of 474 animals, while representing a valuable deep-resequencing cohort for yaks, remains relatively modest for quantitative trait dissection. This constrained sample size inevitably impacts both GWAS statistical power and genomic prediction accuracy. In GWAS, 474 individuals may lack sufficient statistical power to detect ultra-minor effect QTLs under stringent Bonferroni correction. In genomic selection, the limited sample size restricts the precision of marker effect estimations across millions of raw variants, which largely accounts for the modest baseline GBLUP accuracy (R = 0.14 ± 0.07). Furthermore, evaluating marker reduction under strict within-fold cross-validation yielded an unbiased accuracy of R = 0.0991 ± 0.0697, indicating that marker prioritization in small reference sets requires integrating multi-omics functional information to achieve robust accuracy gains. To address these limitations, future research should integrate multi-farm collaborative cohorts and cross-regional reference populations to expand sample sizes and strengthen the foundation for genomic evaluation in indigenous yak populations.

## 5. Conclusions

Using whole-genome resequencing data from 474 Ashidan yaks, this study evaluated the genetic architecture and genomic prediction potential for average daily gain (ADG). Multi-model GWAS identified 11 significant SNP loci associated with ADG, pointing to positional candidate genes such as *PDE10A*, *RAD51B*, and *BCAS3*. Functional analyses indicated potential roles for cAMP signaling, ion channel activity, and extracellular matrix pathways in early growth traits. The estimated narrow-sense genomic heritability (h^2^ = 0.3233) indicates moderate heritability for early ADG. While full-genome prediction showed baseline accuracy (R = 0.14 ± 0.07), evaluating the prioritized top 1% core marker panel under strict 10-fold within-fold cross-validation yielded an unbiased accuracy of R = 0.1328 ± 0.1566 (with an average RMSE of 0.3793 ± 0.0066), reflecting marker effect sampling variance in small cohorts. Overall, these findings offer essential baseline parameters and candidate targets for future genomic selection in indigenous yak populations.

## Figures and Tables

**Table 1:** Genetic Parameters of Ashidan Yak.

Parameters	Estimate	Standard Error (SE)	(*p*-Value)
Additive genetic variance (V_a_)	0.00104	0.00054	
Residual variance (V_e_)	0.00218	0.00052	
Phenotypic variance (V_p_)	0.00322		
Narrow-sense heritability (h^2^)	0.3233	0.1633	0.0478

## Data Availability

The genotypic data used and/or analyzed during the current study are available from the corresponding author on reasonable request.

## Supplementary Materials

The following supporting information can be downloaded at: https://www.mdpi.com/article/10.3390/ani16162452/s1, Figure S1: Manhattan plots (A) and Q-Q plots (B) derived from the genome-wide association analysis of the GLM model for average daily gain in Ashdan yak; Figure S2: Manhattan plots (A) and Q-Q plots (B) derived from the genome-wide association analysis of the MLM model for average daily gain in Ashdan yak; Table S1: Top_1Percent_Chip_Targets; Table S2: Summary of significantly enriched GO terms and KEGG pathways for candidate genes associated with average daily gain (ADG) in Ashidan yaks.

## Abbreviations

The following abbreviations are used in this manuscript:

ADG	Average daily gain
SNP	Single nucleotide polymorphism
GWAS	Genome-wide association study
WGS	Whole-genome sequencing
PCA	Principal component analysis
LD	Linkage Disequilibrium
GO	Gene Ontology
KEGG	Kyoto Encyclopedia of Genes and Genomes
CV	Coefficient of variation
FarmCPU	Fixed and Random Model Circulating Probability Unification
GBLUP	Genomic best linear unbiased prediction
GEBV	Genomic estimated breeding value
GLM	General linear model
GS	Genomic selection
QTL	Quantitative trait locus
