# Supplementary material for: Genome-Wide Association Study and Genomic Selection for Average Daily Gain in Ashidan Yak
Source: Animals (Basel). 2026 Aug 7;16(16):2452. doi: 10.3390/ani16162452 (PMC13508993; doi:10.3390/ani16162452)
Supplement: Supplementary file 1 [file animals-16-02452-s001.zip › Supplementary Figure S2.pdf]

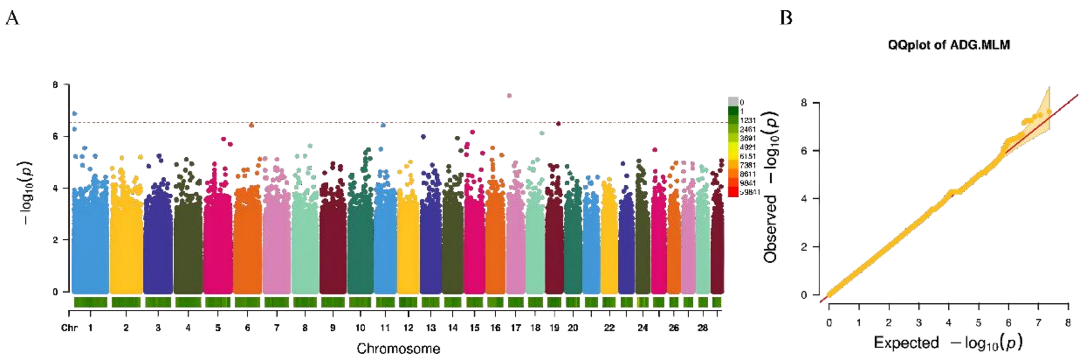

Supplementary Figure S2. Manhattan plots (A) and Q-Q plots (B) derived from the genome-wide association analysis of the MLM model for average daily gain in Ashdan yak.
